# Metabolite-sensing GPCRs controlling interactions between adipose tissue and inflammation

**DOI:** 10.3389/fendo.2023.1197102

**Published:** 2023-07-06

**Authors:** Elaine M. Duncan, Luca Vita, Bethany Dibnah, Brian D. Hudson

**Affiliations:** Centre for Translational Pharmacology, University of Glasgow, Glasgow, United Kingdom

**Keywords:** free fatty acid, succinate, hydroxy carboxylic acids, G protein-coupled receptor, adipose, inflammation, metabolite signalling

## Abstract

Metabolic disorders including obesity, diabetes and non-alcoholic steatohepatitis are a group of conditions characterised by chronic low-grade inflammation of metabolic tissues. There is now a growing appreciation that various metabolites released from adipose tissue serve as key signalling mediators, influencing this interaction with inflammation. G protein-coupled receptors (GPCRs) are the largest family of signal transduction proteins and most historically successful drug targets. The signalling pathways for several key adipose metabolites are mediated through GPCRs expressed both on the adipocytes themselves and on infiltrating macrophages. These include three main groups of GPCRs: the FFA4 receptor, which is activated by long chain free fatty acids; the HCA_2_ and HCA_3_ receptors, activated by hydroxy carboxylic acids; and the succinate receptor. Understanding the roles these metabolites and their receptors play in metabolic-immune interactions is critical to establishing how these GPCRs may be exploited for the treatment of metabolic disorders.

## Introduction

Metabolic disorders, including metabolic syndrome, diabetes and non-alcoholic steatohepatitis (NASH) are a major global health and economic burden, with diabetes alone affecting an estimated 537 million adults globally (1). These conditions are characterised by chronic inflammation infiltrating metabolic tissues, resulting in a disruption to metabolic homeostasis and downstream complications. As obesity is a pertinent risk factor for many metabolic disorders, understanding the pathophysiology of these conditions is of utmost importance given its increasing global prevalence.

The relationship between chronic low-grade inflammation and obesity is well documented. Obesity has been associated with increased macrophage infiltration to adipose tissue in both mouse (2) and human (3). Release of pro-inflammatory cytokines by infiltrating macrophages is enhanced in obesity, contributing to impaired insulin sensitivity (4). The links between inflammation, obesity and insulin resistance have been reviewed extensively elsewhere (5, 6); however, many of the exact mechanisms involved in this interaction are yet to be elucidated.

It is now clear that several metabolic intermediates that are released from adipose tissue play important roles communicating with invading immune cells, in particular macrophages. Several of these metabolites are ligands for G protein-coupled receptors (GPCRs), and, importantly, some of these GPCRs are expressed in both adipocytes and the inflammatory cells that invade metabolic tissues in metabolic disorders (7). GPCRs are the most historically successful drug targets and understanding how these receptors control interactions between metabolism and inflammation may provide new avenues to treat metabolic disorders. Here we identify three metabolic intermediates: long chain fatty acids, hydroxy carboxylic acids, and succinate, that are released either directly or indirectly from adipocytes and signal through GPCRs expressed both on adipocytes themselves and on invading macrophages. In this review we discuss the roles the receptors for these metabolites play controlling interaction between adipocytes and macrophages, as well as how this may contribute to the development, progression, and, ultimately, treatment of metabolic disorders.

## FFA4 free fatty acid receptor

FFA4, formerly GPR120, is a GPCR activated by long chain fatty acids and is reported to have an important role in interactions between metabolism and inflammation. FFA4 is primarily described as a Gα_q_ coupled GPCR, but reports also link it to signalling through Gα_i_ and Gα_s_, and it strongly engages with β-arrestin mediated pathways (8–10). There has been significant interest in FFA4, in part because FFA4^-/-^ mice on a high-fat diet have increased body weight, accumulation of macrophages in their adipose, elevated fasting glucose levels and impaired insulin signalling (11). Furthermore, a deleterious variant of FFA4 (p.R254H/p.R270H) is associated with increased risk of obesity (11) and elevated fasting glucose (12). These findings have generated interested in FFA4 as a therapeutic target for the treatment of inflammatory metabolic disorders.

It is well known that certain long chain fatty acids, in particular the omega (n)-3 fatty acids, possess anti-inflammatory properties, with benefits reported in cardiovascular disease, diabetes, cancer, mental illness and dementia (13). Importantly, the n-3 fatty acids are known to be agonists of FFA4 (14–16). Early evidence also indicated many anti-inflammatory properties of dietary n-3 fatty acids were mediated by FFA4-β-arrestin signalling (17). However, several later studies have failed to reproduce this finding, suggesting instead that dietary n-3 fatty acids are protective regardless of FFA4 expression (18, 19). Therefore, although it is clear that n-3 fatty acids are agonists of this receptor and that dietary n-3 fats provide health benefits, the extent to which FFA4 contributes to the beneficial properties of these fatty acids in the diet remains unclear.

FFA4 is highly expressed in adipose tissue, and its expression increases following *in vitro* adipogenic differentiation of isolated primary human or murine 3T3-L1 adipocytes (20). It has been shown that FFA4 enhances adipogenesis, as its inhibition reduces lipid accumulation and expression of key adipogenic markers (20–22). Interestingly, it has been proposed that in preadipocyte cilia, FFA4 coupling to Gα_s_ to increase ciliar cAMP may be an important trigger for adipogenesis (10). Adipocytes release long chain fatty acids through lipolysis, and it is established that fatty acid levels increase in culture medium during adipogenic differentiation. As these long chain fatty acids released through lipolysis are known agonists of FFA4 (16), this perhaps suggests FFA4 functions as part of a positive autocrine feedback loop reinforcing adipogenic differentiation.

The concept that FFA4 responds to fatty acids released by lipolysis is supported by the observation that conditioned medium from adipocytes treated with a β-adrenoceptor agonist to stimulate lipolysis is sufficient to activate FFA4 receptor signalling *in vitro* (23). In adipocytes, FFA4 couples strongly to the Gα_i_ pathway to reduce cAMP levels (23), a signalling pathway that is well known to inhibit lipolysis (24). Consistent with this, FFA4 has been found to inhibit lipolysis both *in vitro* and *in vivo* (23, 25). These findings indicate FFA4 is part of a negative feedback loop contributing to the long-recognised ability of the fatty acids released from adipocytes to regulate lipolysis (26, 27). FFA4, likely through a Gα_q_ pathway, also enhances GLUT4-mediated glucose uptake in both primary murine adipocytes and 3T3-L1 adipocytes (15, 17). To date, no studies have directly explored whether FFA4 regulation of glucose uptake is controlled in an autocrine fashion by long chain fatty acids released from adipocytes; this is clearly an area that needs future attention.

Chronic low-level inflammation of adipose tissue plays an important role in metabolic syndrome, particularly influencing the development of insulin resistance (4, 28). In immune cells, FFA4 is highly expressed in macrophages (17, 29, 30) and has been recently shown to be expressed in bovine neutrophils where it may play a role in the production of superoxides (31). In RAW 264.7 macrophages, activation of FFA4 through a β-arrestin pathway attenuated pro-inflammatory response to LPS, an effect which was abolished by FFA4-knockdown (17). Furthermore, it has been reported that FFA4 activation reduces macrophage infiltration of adipose tissue (32). Supporting this observation, macrophages displayed chemotaxis toward adipocyte conditioned medium, which was suppressed by FFA4 agonists (17, 32). No subsequent studies have investigated FFA4-regulated infiltration of adipose tissue macrophages, although a role for the receptor in migration is supported by observations that FFA4 activation attenuated the motility of alveolar macrophages (33) and migration of monocytes to atherosclerotic lesions (34). Given that adipocytes release fatty acids through lipolysis, these findings suggest that FFA4 may have an important paracrine signalling role between adipocytes and infiltrating macrophages. Although to date no studies have directly explored this, it will be important to establish if macrophage chemotaxis toward adipocyte conditioned medium is suppressed when the adipocytes are first exposed to a β-adrenoceptor agonists to stimulate lipolysis.

A critical question in understanding FFA4 autocrine or paracrine signalling will be to identify the specific types of fatty acids that are involved. There is evidence that different FFA4 signalling pathways may be activated by specific long chain fatty acids, with a particular difference observed between saturated and unsaturated fatty acids; both classes can activate Gα_i_ and Gα_q_ signalling pathways *in vitro*, however unsaturated fatty acids can also activate the Gα_s_ pathway (35). Recent lipidomics data suggests that unstimulated mouse and human adipocytes typically release saturated fatty acids (stearic & palmitic acids), with an increase in unsaturated fatty acid release (linoleic & oleic acids) following β-adrenoceptor stimulated lipolysis (23). This may therefore indicate that the fatty acids released through basal adipocyte lipolysis will produce different FFA4 signalling profiles than the fatty acids released due to adrenergic stimulated lipolysis. Similarly, it will also be important to establish whether specific fatty acids from alternative sources, for example released from macrophages by phospholipase A2 (PLA_2_) hydrolysis of phospholipids, have a role modulating adipocytes/macrophages communications through FFA4. The main products of PLA_2_ hydrolysis are poly unsaturated n-3 and n-6 fatty acids, which are all known to activate FFA4 (16). Secreted PLA_2_ is expressed in adipose tissue macrophages, and the activity of PLA_2_ protects mice from diet induced obesity and adipose inflammation (36, 37). However, the potential for unsaturated fatty acids released by macrophage PLA_2_ contributing to autocrine or paracrine FFA4 signalling in adipose has not yet been investigated.

There has also been interest recently in the role of FFA4 in the progression and treatment of NASH. FFA4 has been suggested to have a protective role in NASH, as supplementation with omega-3 fatty acids reduces liver inflammation in an FFA4-dependent manner (38, 39). Furthermore, in an inflammatory mouse model of NASH, FFA4 appears to be upregulated in white adipose tissue and reduces the expression of pro-inflammatory IL-6 and TNF-α in this tissue (40). This may be particularly important therapeutically, given that FFA4 is also anti-lipolytic and thus may provide benefit to both the lipotoxity and inflammatory aspects of NASH.

**Figure 1:**
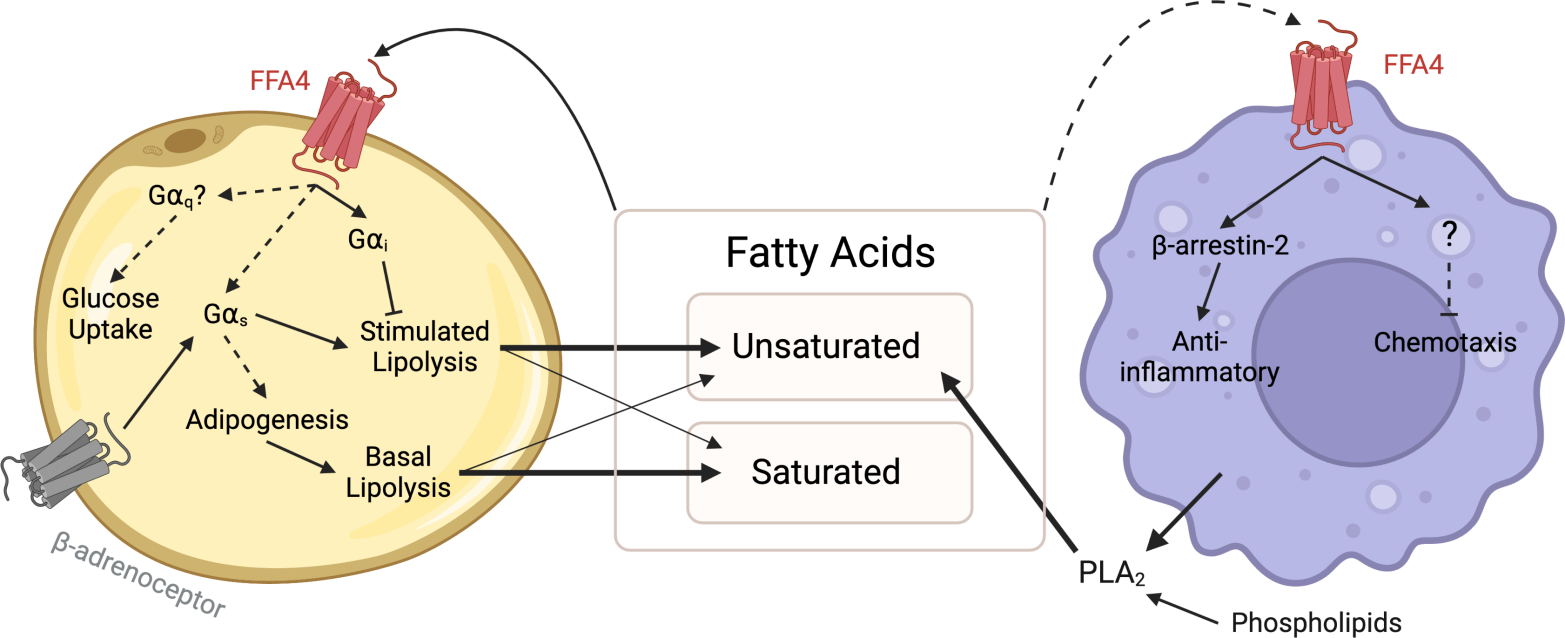
FFA4 signalling between adipocytes and macrophages. Saturated and unsaturated fatty acids are released from adipocytes by lipolysis, supplemented from the diet and may be produced through PLA_2_-mediated hydrolysis of phospholipids. These fatty acids act as ligands of FFA4 in an autocrine manner to inhibit lipolysis *via* Gα_i_ signalling, stimulate glucose uptake *via* a Gα_q_ pathway and stimulate or enhance adipogenesis *via* Gα_s_ signalling. Fatty acids may also act in a paracrine manner to activate anti-inflammatory pathways in macrophages. Dashed lines represent interactions lacking direct mechanistic evidence, and thus further investigation is required. Created with BioRender.com.

Overall, there is substantial evidence that FFA4 is involved in the interaction between metabolism and inflammation and likely is a central player directly mediating communication between adipocytes and macrophages (**Figure 1**). However, our current lack of mechanistic understanding for how FFA4 signalling operates under physiological and pathophysiological conditions needs to be resolved before we can truly establish if this receptor is a viable target for metabolic disease.

## Hydroxyl carboxylic acid receptors

The hydroxy-carboxylic acid (HCA) family of receptors form a cluster of class A GPCRs, which share significant sequence identity. The family includes three known receptors, HCA_1_, HCA_2_ and HCA_3_, formerly known as GPR81, GPR109A and GPR109B respectively (41). The receptors are activated by various different hydroxy-carboxylic acid metabolites whose plasma concentrations vary depending on the metabolic state of the organism, suggesting the receptors play a role in metabolic homeostasis. The HCA receptors are all expressed in adipose tissue and respond either directly or indirectly to metabolites released from adipocytes (42). In adipocytes all three receptors couple to Gα_i_ to mediate anti-lipolytic effects *via* inhibition of cAMP (43), while HCA_2_ in particular has also been reported to signal through β-arrestins in some contexts (44) as well as through Gβγ subunits specifically in macrophages (45). Only HCA_2_ and HCA_3_ receptors are expressed on certain immune cells, including macrophages and neutrophils, suggesting these two receptors may have a role modulating inflammation in response to metabolic signals released from adipocytes.

The HCA_2_ receptor was first described as a receptor for the nutrient, nicotinic acid, in 2003, with evidence showing nanomolar affinity for the receptor (46, 47). Nicotinic acid has long been known to produce an antilipolytic effect in adipose, resulting in decreased plasma FFA concentrations (48). This effect of nicotinic acid is abolished in HCA_2_ knockout mice (46). However, as endogenous concentrations of nicotinic acid in the body are too low to activate the receptor, it is unlikely that this is the endogenous ligand of HCA_2_ (49). Instead, a second small carboxylic acid with activity at HCA_2_ has been identified, the ketone body β-hydroxybutyrate (BHB) (49). Like nicotinic acid, previous research had demonstrated BHB to have anti-lipolytic effects (50–52), which were found to be dependent on HCA_2_ (49). Critically, despite the fact that BHB only has high micromolar affinity for HCA_2_ (49), plasma concentrations of BHB increase to low millimolar levels during fasting (53–56), suggesting that BHB is a legitimate endogenous ligand of HCA_2_. The source of elevated BHB during fasting, involves first the lipolytic release of FFAs from adipose, followed by oxidation of these fatty acids in the liver to produce BHB (57–59). Given the anti-lipolytic effects of HCA_2_, this suggests a primary role for the receptor in a negative feedback loop to regulate lipolytic rate and preserve energy during fasting (60). Interestingly, a recent study has found that adipocytes also have the ability to produce and secrete BHB (61), suggesting that HCA_2_ signalling could also be activated directly through local autocrine or paracrine signalling in adipose, as well as the more traditional indirect activation *via* BHB production in liver.

HCA_2_ is highly expressed in both human and murine white and brown adipose tissue, with expression increasing through adipogenesis of common adipocyte cell models (62, 63). To a lesser extent the receptor is also expressed in macrophages, with evidence showing that HCA_2_ expression is upregulated in the presence of proinflammatory stimuli, like lipopolysaccharide (LPS) and TNF-α (64, 65). A similar increase in HCA_2_ expression in response to LPS is also observed in cultured adipocyte models *in vitro* (66). In contrast, adipocyte HCA_2_ expression decreases in diet induced obese mice (66), and expression is decreased in subcutaneous adipose tissue of obese human subjects (67). These contrasting *in vitro* and *in vivo* findings may suggest that simple treatment of *in vitro* adipocytes with pro-inflammatory mediators does not accurately reproduce the chronic multicellular inflammatory responses observed in obese adipose (68). This highlights a need to develop more accurate *in vitro* approaches to allow for thorough investigation and to better understand how inflammation affects HCA_2_ expression and function.

The HCA_3_ receptor is closely related to HCA_2_, sharing 96% sequence identity. It is the result of a gene duplication that is present only in human and hominids (69). HCA_3_ has a very similar expression pattern to HCA_2_, being highly expressed in adipocytes, as well as several immune cells (47, 70). Despite the high level of similarity, the receptors do not share the same endogenous ligand. HCA_3_ is not activated by BHB, but instead by 3-hydroxyoctanoate (71). Like BHB, 3-hydroxyoctanoate is produced in the liver and muscles by β-oxidation of fatty acids produced by lipolysis and so HCA_3_ also appears to act as a negative feedback modulator of lipolysis during fasting (72). Like HCA_2_, addition of LPS has been found to significantly increase the expression of HCA_3_ in adipocytes and macrophages cultured *in vitro* (63).

Due to the increase in HCA_2_ and HCA_3_ expression with LPS treatment, recent studies have investigated whether these receptors play a role in modulating proinflammatory cytokine production. Activation of HCA_2_ and HCA_3_ with nicotinic acid and 1-isopropyl-1H-benzotriazole-5-carboxylic acid (IPBT) respectively, reduced the production of proinflammatory cytokines in SGBS adipocytes and THP-1 macrophages exposed to LPS (63). Likewise, HCA_2_ activation in primary murine macrophages had the same effect (73). Inflammatory cytokines have a key role in the development of metabolic syndrome, disrupting insulin and lipid signalling pathways (74). In addition, activation of HCA_2_ also has been found to suppress signalling responses in macrophages to key chemoattractant chemokines, CCL2, fMLF and RANTES (45, 75). Critically, these chemokines have important roles in the macrophage infiltration of adipose in metabolic disease (76). Together, this suggest HCA_2_ and HCA_3_ may be important regulators of inflammation in adipose tissue and potential targets for the treatment of metabolic disorders.

Interestingly HCA_2_ signalling differs in macrophages and adipocytes. In macrophages, although the receptor still couples to Gα_i_, this coupling results in increased intracellular calcium (likely *via* activation of Gβγ subunits) and is associated with the release of prostanoids (45). The HCA_2_ mediated release of prostanoids also appears to involve β-arrestin-1, as macrophages from β-arrestin-1^-/-^ mice show reduced activation of cytosolic phospholipase A2, the first step in prostanoid release, when treated with an HCA_2_ agonist (44). Interestingly, prostanoid release from macrophages also seems to serve as an autocrine regulator for HCA_2_ signalling, resulting in an unexpected HCA_2_ mediated increase in cAMP levels through a Gα_i_-Gβγ pathway (45). To date the importance of this pathway has been explored only in relation to antiatherosclerotic properties of HCA_2_, and so it will be critical to establish what impact this pathway has on the regulation of adipose inflammation by HCA_2_.

**Figure 2:**
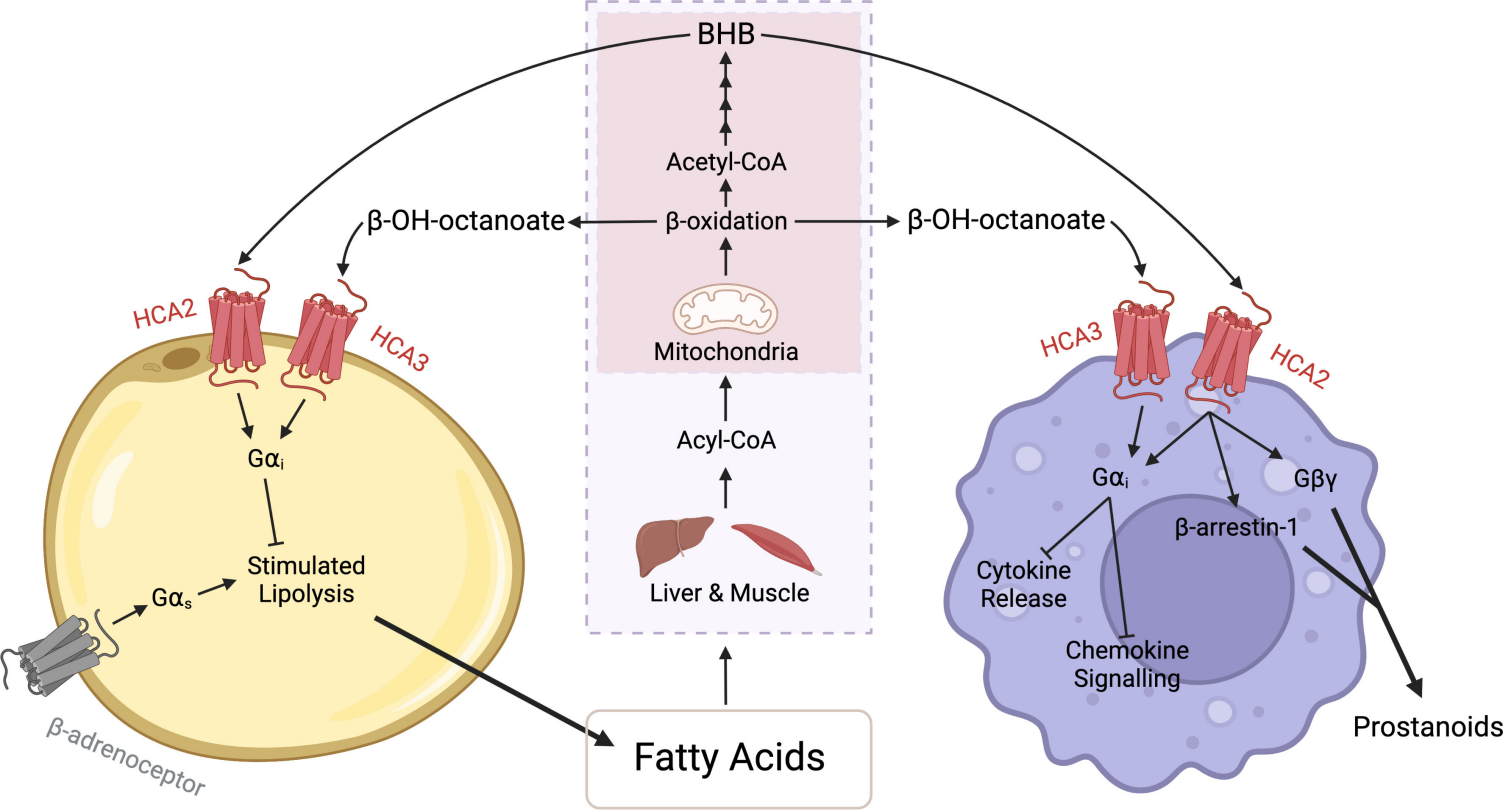
HCA receptor signalling between adipocytes and macrophages. During periods of fasting or exercise, there is an increase in lipolysis and subsequently an increased release of fatty acids from adipocytes. These fatty acids are converted in the liver or muscle (purple box) to Acyl-CoA and then transported into mitochondria (red box). Beta-oxidation in the mitochondria produces acetyl-CoA, which undergoes ketogenesis, a four-step process that can produce beta-hydroxybutyrate (BHB). BHB binds to and activates the HCA_2_ receptor and through Gα_i_ signalling, evokes antilipolytic effects in adipocytes and anti-inflammatory effects in macrophages. The anti-inflammatory effects include a reduction in cytokine production and suppression of chemokine signalling. Additionally, in macrophages the HCA_2_ receptor signals *via* the Gβγ subunit and β-arrestin-1 to mediate the release of prostanoids. Similarly, beta-hydroxyoctanoate is produced *via* beta-oxidation of fatty acids. However, beta-hydroxyoctanoate is released as a result of incomplete beta-oxidation. Beta-hydroxyoctanoate binds to and activates the HCA_3_ receptor on adipocytes and macrophages and through its Gα_i_ evokes anti-inflammatory and antilipolytic effects. Created with BioRender.com.

The HCA receptors play an important role in controlling metabolic homeostasis through an anti-lipolytic negative feedback loop, while also playing an immunomodulatory role (**Figure 2**). Previously, production of endogenous HCA ligands was thought to occur exclusively in the liver and muscle, however recent research suggests that adipocytes are also capable of directly secreting certain HCA receptor ligands. It will be important for future work to address what, if any, contribution adipose secreted HCAs have to overall HCA receptor signalling, and how these receptors contribute to adipose-immune cell communication.

## Succinate receptor

GPR91 was identified in 2001 as an orphan GPCR sequence located on chromosome 3 with homology to purinergic receptors (77). Subsequent work demonstrated that GPR91 is activated by the citric acid cycle intermediate, succinate (78), and the receptor was subsequently renamed the succinate receptor, but is also commonly referred to by its gene name, SUCNR1 (79). Initial expression studies found high levels of SUCNR1 in kidney, liver and spleen (78), while the receptor has also been found in adipose (80), and in various immune cells including dendritic cells and macrophages (81). Since its discovery, SUCNR1 has received significant attention for its role in the pathophysiology of, and potential treatment for, a variety of conditions including hypertension, cardiovascular disease, obesity and insulin resistance, NASH, macular degeneration and inflammatory bowel diseases (82).

Although succinate is normally found primarily in the mitochondrial matrix, under conditions of hypoxic and metabolic stress, succinate dehydrogenase, which converts succinate to fumarate as part of the citric acid cycle, reverses its function, resulting in a build-up of succinate in the mitochondria (83). This excess succinate is transported out of the mitochondria through a mitochondrial dicarboxylic acid carrier (84), before ultimately being released from the cell through solute carrier transporters (85). Extracellular succinate is then able to bind to and activate SUCNR1. While this process occurs in many cell types, it may be particularly important in adipocytes, which become hypoxic with chronic low-level inflammation in obesity, metabolic syndrome and diabetes (86).

SUCNR1 is primarily described as a Gα_i_ coupled GPCR inhibiting production of cAMP, while a few studies have also observed Gα_q_ mediated SUCNR1 signalling (78, 87, 88). With this in mind, SUCNR1 has been shown to inhibit adipocyte lipolysis both *in vitro* and *ex vivo* in a Gα_i_ dependent manner (80). Subsequent studies have also demonstrated anti-lipolytic effects of SUCNR1 *in vivo* (89), and specifically linked these effects to adipocyte expression of a dicarboxylic acid carrier responsible for transporting succinate out of mitochondria (84). Together, these findings support an autocrine signalling pathway mediated by succinate released from adipocytes to control lipolysis.

Mice lacking SUCNR1 have a variety of disfunctions in their metabolic phenotype when fed a high fat diet, including: hyperglycemia, reduced weight gain, and impaired glucose clearance (89). The fact that these metabolic phenotypes in SUCNR1^-/-^ mice are only observed with a HFD, suggests a need for metabolic stress and/or hypoxia in the adipose for SUCNR1 pathways to be active. Indeed, there is clear evidence that plasma succinate levels are elevated in human patients with obesity, metabolic syndrome and diabetes (90–92). Given that each of these conditions is associated with chronic inflammation of adipose, and that SUCNR1 is also expressed in immune cells, this implicates SUCNR1 as a potential mediator of adipocyte-immune cell communication. Providing support for this possibility, abnormal metabolic phenotypes have been observed in mice with a myeloid specific SUCNR1 knockout (93), suggesting a key role of SUCNR1 in macrophages metabolic dysfunction.

While there is now clear evidence that succinate-SUCNR1 signalling plays an important role mediating communication between adipose tissue and macrophages, it remains controversial whether SUCNR1 is pro- or anti-inflammatory. Studies in SUCNR1^-/-^ macrophages have indicated that this receptor mediates chemotaxis towards succinate released from hypoxic adipocytes (90), suggesting a pro-inflammatory function. This is consistent with earlier work reporting a pro-inflammatory role of SUCNR1 in macrophages in various other inflammatory disorders, including arthritis (94), arthrosclerosis (95), and inflammatory gut conditions (96, 97). In contrast, others have reported pro-inflammatory cytokine secretion to be enhanced in SUCNR1^-/-^ macrophages (98), and adipose tissue inflammation to be exacerbated in myeloid cell specific SUCNR1 knockout mice fed a high fat diet (93), suggesting an anti-inflammatory role of the receptor. Consistent with this, a recent transcriptomic study found that succinate, acting through an SUCNR1-Gα_q_ pathway, hyperpolarized human macrophages toward the M2 anti-inflammatory phenotype (87). Adding a further layer of complexity, studies in adipose taken from lean vs obese patients found that while succinate was anti-inflammatory in the lean adipose, it enhanced IL-1β and TNF expression in obese adipose (93), suggesting cell or context dependent SUCNR1 control of inflammation. Developing a clear understanding of the role of SUCNR1 in inflammation will be key to establishing whether agonism or antagonism of the receptor will have therapeutic benefit.

In addition to the release of succinate from hypoxic adipocytes, macrophages themselves also directly release succinate in response to inflammatory signals (99). While there is evidence that succinate released from macrophages does act in an autocrine fashion through SUCNR1 to affect inflammatory responses (94), to date it is not clear whether macrophage released succinate mediates communication to adipocytes through this receptor. In addition, circulating succinate levels are utilised and cleared by uncoupling protein 1 (UCP1) expressed in brown and beige adipose (100, 101), and genetic disruption of UCP1 leads to SUCNR1 mediated liver inflammation (100). This raises interesting questions about the potential use of SUCNR1 in treating liver diseases like NASH, given that, like many other receptors that inhibit lipolysis in adipose including both FFA4 and HCA_2/3_, SUCNR1 actions in adipose have protective effects on liver lipotoxicity (84). This indicates that like its control of inflammation more broadly, the potential of SUCNR1 in treating metabolic and inflammatory mediated liver disease is complex.

**Figure 3:**
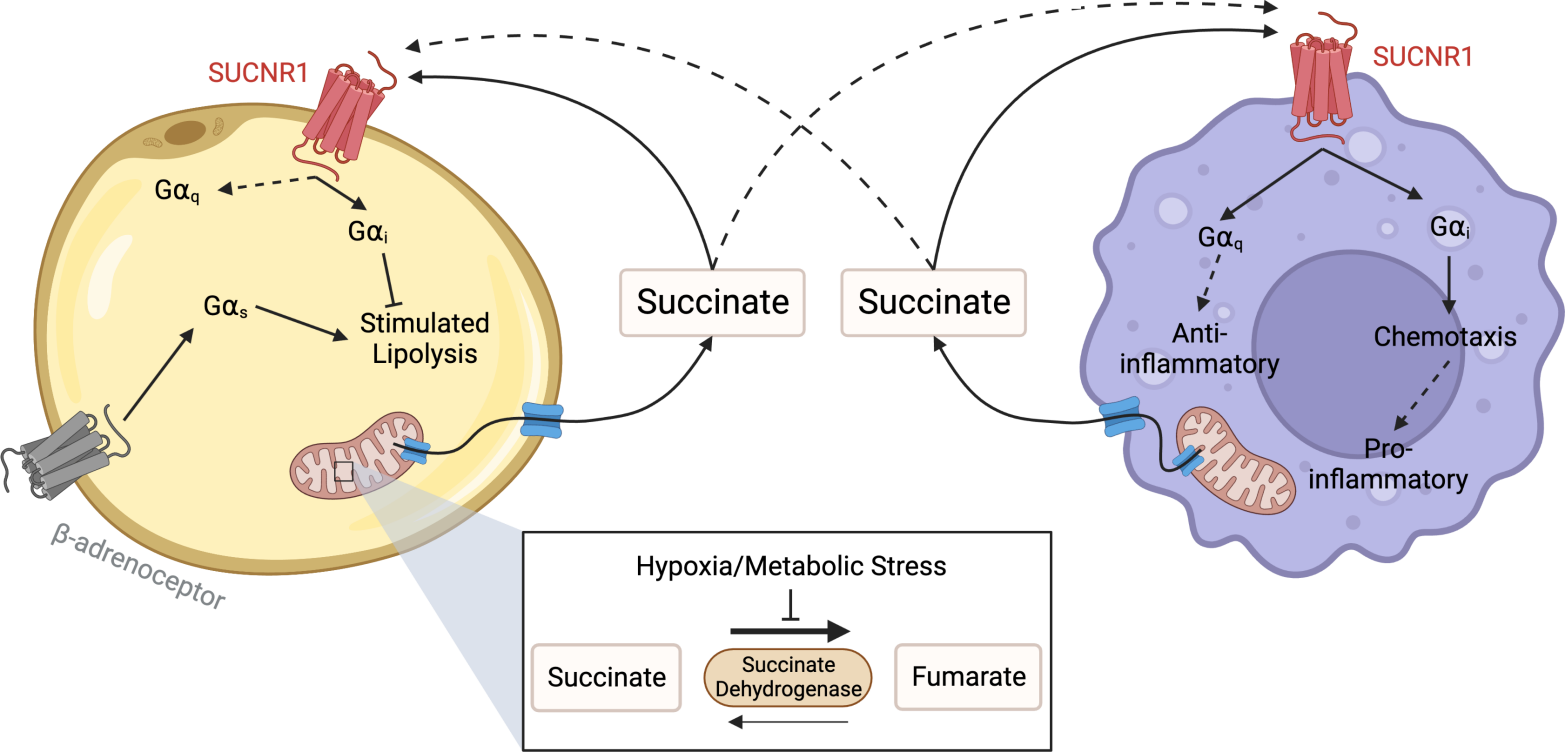
Succinate Receptor signalling between adipocytes and macrophages. Under conditions of hypoxic or metabolic stress succinate dehydrogenase reverses function, leading to a build-up of succinate in the mitochondria. When concentrations become elevated, succinate leaves the mitochondria through a dicarboxylate carrier, before exiting the cell through other solute carriers. Once outside the cell, succinate binds to and activates SUCNR1 on the surface of adipocytes and macrophages to inhibit lipolysis and control inflammation respectively. In macrophages, SUCNR1 has been reported to have both pro-inflammatory and anti-inflammatory effects. These include mediating chemotaxis towards hypoxic adipose, likely through Gα_i_ activation, as well as hyperpolarisation to the M2 macrophage phenotype through a Gα_q_ mediated pathway. Created with BioRender.com.

SUCNR1 signalling has an important role in regulating metabolism and inflammation in the context of metabolic disease (**Figure 3**). Succinate, acting through SUCNR1, is an important mediator of stress signalling to affect immune cell function and inflammation. However, given the conflicting findings on this receptor as either pro- or anti-inflammatory, there is a real need to establish more physiologically relevant approaches to better understand the role of SUCNR1 and determine if and how we can target this receptor for the treatment of disease.

## Conclusion

Various metabolites released by adipocytes act through either direct or indirect signalling pathways to activate metabolite-sensing GPCRs both in adipocytes themselves and in the immune cells that infiltrate adipose in metabolic disorders. These receptors play a variety of functional roles, but commonly both regulate inflammation in immune cells and lipolytic pathways in adipose. The complex, multicellular nature of these signalling pathways and networks has made mechanistically dissecting this signalling quite difficult *in vitro*, and it will be critical to develop and establish more robust experimental models and approaches to achieve this.

Not surprisingly, given the functions of the FFA4, HCA and SUCNR1 receptors in controlling metabolism and inflammation, each of these has received interest as a potential target in the treatment of metabolic disorders including dyslipidaemia, diabetes and NASH. HCA_2_ is the most well developed, with its naturally occurring ligand, niacin, having been widely used clinically to control dyslipidaemia through a mechanism that is at least partly mediated by HCA_2_ (102). However, these clinical studies have also demonstrated that niacin, also *via* HCA_2_, produces an unwanted flushing effect in skin (103). Efforts to eliminate this side effect led to the development of a synthetic partial HCA_2_ agonist, MK-0354 that maintains lipid lowering effects, but without producing flushing in animal models (104). These findings led to MK-0354 entering phase I and II clinical trials, where although it did not produce flushing, it also did not improve lipid levels following chronic treatment (105). Despite this failure, medicinal chemistry efforts have continued around HCA_2_, but without any yet reaching the clinic (106). Similarly, FFA4 has received significant attention from both academic and industrial drug discovery programmes, primarily focused on developing agonists for type 2 diabetes and/or NASH, however to date no molecules have entered the clinic (107). While also receiving some attention as a therapeutic target, the conflicting data around the benefits of agonism vs antagonism of SUCNR1, as well as the relatively wide spread expression pattern for SUCNR1 in multiple tissues and cell types (82) has perhaps led to a somewhat slower pace of development for this receptor.

Ultimately, understanding the signalling networks of these metabolite-sensing GPCRs will help us better understand how interactions between metabolism and inflammation drive metabolic disease. Developing this understanding is likely to open new opportunities for the treatment of a variety of metabolic disorders, including obesity, diabetes and NASH.

## Author contributions

All authors listed have made a substantial, direct, and intellectual contribution to the work and approved it for publication.
